# Serum glutamate dehydrogenase activity enables early detection of liver injury in subjects with underlying muscle impairments

**DOI:** 10.1371/journal.pone.0229753

**Published:** 2020-05-14

**Authors:** Shelli Schomaker, David Potter, Roscoe Warner, Jane Larkindale, Nicholas King, Amy C. Porter, Jane Owens, Lindsay Tomlinson, John-Michael Sauer, Kent Johnson, Jiri Aubrecht

**Affiliations:** 1 Drug Safety R&D, Pfizer, Inc, Groton, CT, United States of America; 2 University of Michigan, Ann Arbor, MI, United States of America; 3 Critical Path Institute, Tucson, AZ, United States of America; 4 Rare Disease Research Unit, Pfizer, Inc, Cambridge, MA, United States of America; University of Navarra School of Medicine and Center for Applied Medical Research (CIMA), SPAIN

## Abstract

Serum activities of alanine and aspartate aminotransferases (ALT and AST) are used as gold standard biomarkers for the diagnosis of hepatocellular injury. Since ALT and AST lack liver specificity, the diagnosis of the onset of hepatocellular injury in patients with underlying muscle impairments is severely limited. Thus, we evaluated the potential of glutamate dehydrogenase (GLDH) as a liver specific alternative biomarker of hepatocellular injury. In our study, serum GLDH in subjects with Duchene muscular dystrophy (DMD) was equivalent to serum GLDH in age matched healthy subjects, while serum ALT was increased 20-fold in DMD subjects. Furthermore, serum GLDH in 131 subjects with variety of muscle impairments was similar to serum GLDH of healthy subjects while serum ALT corelated with serum creatine kinase, a widely accepted biomarker of muscle impairment. In addition, significant elevations of ALT, AST, and CK were observed in a case of a patient with rhabdomyolysis, while serum GLDH stayed within the normal range until the onset of hypoxia-induced liver injury. In a mouse model of DMD (*DMD*^*mdx*^), serum GLDH but not serum ALT clearly correlated with the degree of acetaminophen-induced liver injury. Taken together, our data support the utility of serum GLDH as a liver-specific biomarker of liver injury that has a potential to improve diagnosis of hepatocellular injury in patients with underlying muscle impairments. In drug development, GLDH may have utility as a biomarker of drug induced liver injury in clinical trials of new therapies to treat muscle diseases such as DMD.

## Introduction

Serum activities of alanine and aspartate aminotransferases (ALT and AST) are used as gold standard biomarkers for the diagnosis of hepatocellular injury. In hepatocytes, both enzymes are present in cytoplasm and released into the circulation when the integrity of the hepatocellular membrane is disrupted. Thus, ALT and AST are highly sensitive biomarkers of liver damage, widely used to diagnose liver disease and/or monitor liver toxicity of drugs and chemicals. However, ALT and AST increases are not specific to liver injury. Since considerable amounts of ALT and AST are present in myocytes, serum ALT and AST activities also increase as a consequence of muscle injury. In fact, persistent transaminasemia is frequently misdiagnosed as liver injury in patients with inherited muscle disorders such as Duchenne muscular dystrophy (DMD) and idiopathic inflammatory myopathies [1–3].

Furthermore, the lack of liver specificity of ALT and AST prevents the detection of the onset of liver injury in subjects with concomitant muscle injury. This deficit can negatively impact and slow the development of new therapies for a variety of muscle diseases such as DMD. Additionally, several widely used therapeutics, such as statins and fibrates, are known to cause muscle toxicity. When these drugs are taken concomitantly with experimental agents in trial subjects, the interpretation of increases in serum ALT, particularly those that are relatively small and transient, can be extremely challenging.

In light of the challenges with these approaches to improve the diagnosis of the onset of liver injury in subjects with underlying muscle diseases, the potential of glutamate dehydrogenase (GLDH) as a liver specific biomarker of hepatocellular injury was evaluated in the current study. GLDH is a mitochondrial enzyme that serves as an interface between carbohydrate and amino acid metabolism [4]. GLDH is primarily found homogenously expressed throughout the liver lobule [5,6] and to a lesser degree in the kidney, pancreas, brain and intestine, with only a trace amount found in muscle tissue [4,7]. Similar to ALT, the GLDH enzyme leaks from the damaged hepatocyte into the circulation upon the loss of hepatocellular membrane integrity and is easily detected *via* an enzymatic activity assay [8]. While both GLDH and ALT are rapidly released from the liver following hepatocellular injury, the elimination half-life of GLDH in serum is 16–18 hours [4,9], while ALT has a half-life in serum of 47 hours [10,11].

In preclinical studies, GLDH has been shown to be similar to or to outperform ALT in terms of both sensitivity and specificity [12–16]. In veterinary practice, GLDH has been used as a biomarker of liver injury in a wide range of species including companion animals, birds and domestic animals [17,18]. In patients hospitalized for acetaminophen (APAP) overdose, GLDH was demonstrated to strongly correlate with ALT to detect acute liver injury [19,20]. Studies have also shown that even mild hepatocellular injury caused by exposure to heparins or cholestyramine can be successfully detected by serum GLDH [21,22]. The most comprehensive evaluation to date of GLDH in healthy subjects and subjects with a variety of liver impairments included over 700 subjects [20]. In this study, GLDH levels were shown to be unaffected by age or gender and a reference range of 1-10U/L was established. In subjects with a variety of liver impairments, GLDH and ALT were highly correlated, yielding a Spearman’s rank correlation coefficient of r_s_ = 0.88. GLDH was also shown to have a high diagnostic power for the detection of liver injury with an area under the receiver operating characteristic (ROC) curve of 0.98 for a broad range of clinically demonstrated liver impairments including APAP-induced liver injury.

In the current study we provide experimental evidence supporting GLDH as a specific biomarker of liver injury that is not affected by either acute or chronic muscle diseases. Furthermore, the ability of GLDH to detect the onset of liver injury in subjects with underlying muscle disease is shown in a case of rhabdomyolysis and in a mouse model of DMD.

## Materials and methods

### Acquisition of samples from healthy subjects

Blood samples from 125 healthy subjects were collected from the University of Michigan health care system (UM) under an approved IRB (HUM0044422). Researchers did not have access to any potentially identifying personal information. The University of Michigan IRB committee waived the requirement for informed consent for this sample-set collection. Samples were defined as healthy based on normal levels of ALT, AST, alkaline phosphatase (ALP), total bilirubin (TBil), glucose, blood urea nitrogen, serum creatinine and CK. Subjects whose values for one or more of the above endpoints exceeded the normal reference range were not used in this study. In addition, any healthy subject that had an ongoing health problem or immunological flare was omitted from the cohort. Most samples were collected from subjects who were at the University of Michigan for routine health examinations.

### Acquisition of samples from subjects with muscle injury

Blood samples from 131 subjects with muscle injury were collected from the University of Michigan health care system (UM) under an approved IRB (HUM0044422). Researchers did not have access to any potentially identifying personal information. J.K. provided medical adjudication of subjects from UM and had access to medical records as required by UM. The University of Michigan IRB committee waived the requirement for informed consent for this sample-set collection. Samples featured abnormal CK enzyme activity levels or clinically demonstrable muscle injury as assessed by medical adjudication. Clinically determined injuries could include, but were not limited to, primary disorders of muscle (dystrophies, myotonic disorders, congenital myopathies and mitochondrial myopathies) and toxic myopathies (drug, alcohol and toxicants), as exhibited by myositis (inflammatory muscle injury), neurogenic atrophy, necrotizing inflammatory muscle injury, chronic severe atrophy, angulated atrophic fibers (AAF), type II fiber atrophy, nuclear myobags, denervation atrophy, and/or increased lipids in myofibers. Additional comorbidities were often present. Samples from patients with liver injury were excluded from this analysis since GLDH would be expected to be elevated in these subjects due to the liver injury. For this analysis, subjects with liver injury based on the clinical chemistry criteria [≥ 5x ALT or ≥ 2x ALP or (≥ 3x ALT or ≥ 2x Tbil)] [23] or evidence of liver injury in the medical records were excluded.

### Acquisition of samples from subjects with DMD

Forty blood samples were collected from patients with DMD seen at the University of Florida under an approved IRB (176–2010) for this evaluation with informed consent and the researchers did not have access to any potentially identifying personal information. These subjects were all ambulatory males (ages 5–14) previously diagnosed with DMD based on 1) clinical features with onset of symptoms before age five, 2) elevated CK levels and 3) absence of dystrophin expression as determined by immunostaining or western blot and/or genetic testing for confirmation of a dystrophin mutation.

### Experimental animals

Male C57BL/10 DMD/J mice (approximately five to six weeks of age) were purchased from the Jackson Laboratory (Bar Harbor, ME). These mice (*DMD*^*mdx*^) have a point mutation in their *DMD* gene that causes their muscle cells to produce a small, nonfunctional dystrophin protein. As a result, the mice have a mild form of DMD where there is increased muscle damage and weakness with elevated serum CK, an early marker of muscle degeneration. Mice were housed in polycarbonate cages on a 12-h light-dark cycle and provided standard diet and reverse osmosis water *ad libitum* throughout the entire course of the study. This study was carried out in strict accordance with the recommendations in the Guide for the Care and Use of Laboratory Animals of the National Institutes of Health. The protocol was approved by the Pfizer Committee on the Ethics of Animal Experiments under Animal Use Protocol 2013-KSQ-00910 and all efforts were made to minimize animal suffering. No anesthesia or analgesia was used. There was no need to alleviate any suffering as it was not expected or encountered, but the protocol allows for pain relief treatments as indicated by a vet, or humane euthanasia in the case of suffering. Carbon dioxide (CO_2_) overdose was used for euthanasia.

### *In vivo* mouse study

Mice (7 per group) were fasted for 5–6 hours and then given a single dose of 300 mg/kg APAP, a model hepatotoxicant, or 0.9% saline by intraperitoneal (i.p.) injection [24]. Blood was collected from each animal at necropsy for assessment of ALT, AST, GLDH and CK. Liver and muscle tissues were collected for histopathological examination. Briefly, the tissues were immersion-fixed for 24hrs in 10% neutral buffered formalin, processed routinely to paraffin block, and 4–5 micron thick slide mounted histologic sections were stained with hematoxylin-eosin. Liver histopathology was analyzed by an American College of Veterinary Pathologists (ACVP) Board Certified Pathologist.

### Sample preparation and analysis

Whole blood was centrifuged at 3000 x g for 10 minutes at room temperature and then serum samples were recovered from serum-separator tubes (SST). Serum samples were frozen at -80°C and stored until shipped for biomarker analysis. GLDH in human serum demonstrates acceptable stability at room temperature up to 48 hours, refrigerated up to 14 days, and frozen at -80 up to 18 months. GLDH in human serum demonstrates acceptable stability for 4 freeze thaw cycles. All samples were analyzed with in the stability windows. As the GLDH assay has been validated, and its robustness and reproducibility demonstrated, duplicate analysis of test samples were not performed.

The Randox GLDH assay was validated according to Centers of Medicare and Medicaid Services’ (CMS) Clinical Laboratory Improvement Amendments (CLIA) guidelines for Laboratory Developed Tests (LDT). As required by CLIA, the laboratory determined performance specifications and was responsible for the quality of the results generated from the test. During assay validation the following parameters were tested: accuracy, precision, analytical sensitivity (Limit of blank (LOB)), long term stability, freeze/thaw stability, analytical specificity to include interfering substances, reportable range, and reference interval. Appropriate quality control samples were applied during the validation procedure and throughout the subsequent sample analysis to ensure data reliability and data comparability over the different clinical studies included in this package. The Randox GLDH assay was also validated across rat, dog, and mouse serum.

For all samples, GLDH was measured in serum on the Siemens ADVIA 1800 or ADVIA 2400 Automated Chemistry System with a commercially available kit (Randox Labs Ltd, Roche). Similarly, all other analytes were measured on the Siemens instruments using commercially available kits (Siemens).

## Results

The lack of liver specificity of ALT prevents the diagnosis of the onset of liver injury in subjects with muscle disease. Since only trace amounts of GLDH are present in muscle tissue, we evaluated this marker as an alternative liver specific biomarker to ALT. To assess the impact of underlying muscle impairment on serum GLDH levels in human subjects, we examined GLDH activity in serum samples from adult subjects with acute and chronic muscle impairments and pediatric patients with DMD, a chronic muscle disease that exhibits extensive muscle injury in the absence of liver injury.

**Fig 1:**
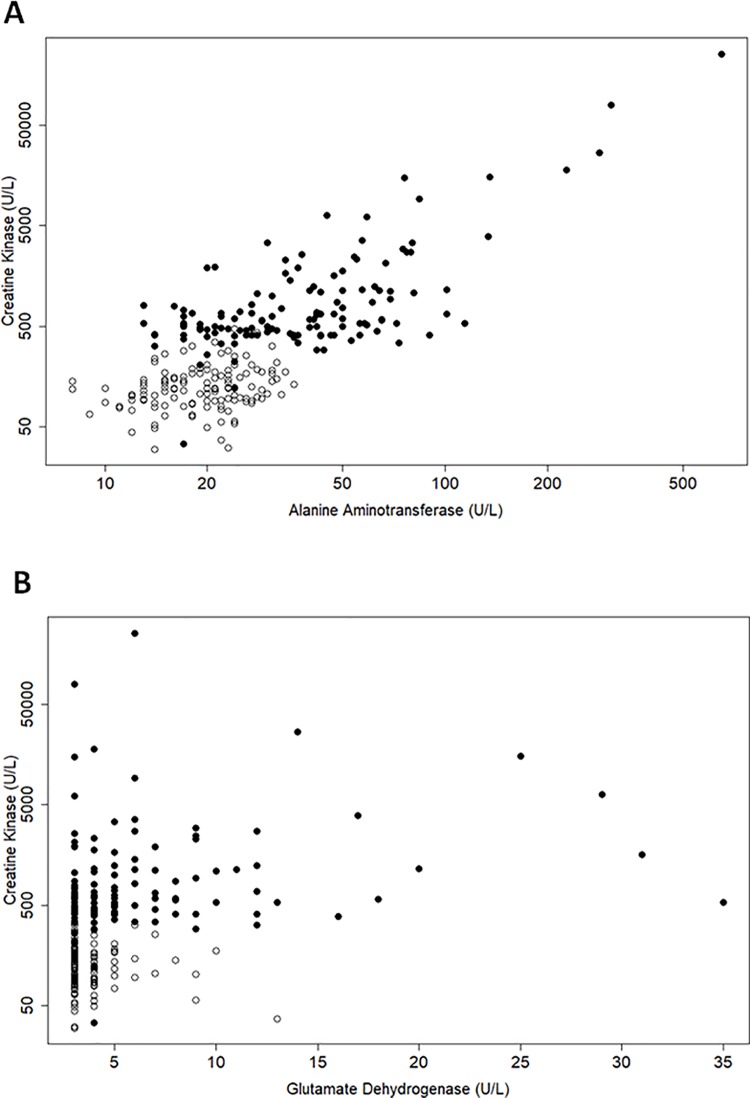
GLDH levels are not affected by acute muscle diseases. A is a comparison of CK and ALT; B is a comparison of CK and GLDH. Each point represents CK, ALT or GLDH values from an individual serum sample collected from a total of 125 healthy subjects (open circles) and 131 subjects with a variety of muscle impairments (closed circles).

Serum from healthy subjects, and subjects with muscle injury with abnormal CK enzyme activity levels and clinically demonstrable muscle injury as assessed by medical adjudication, were prospectively selected. A total of 256 samples from 125 healthy subjects and 131 subjects with varying degrees of muscle impairment were analyzed. All but four subjects had GLDH levels <2.5x ULN (upper limit of normal) indicating a lack of liver injury. A reason for the high GLDH levels in one of these four subjects could not be elucidated from their medical records. Higher GLDH levels in the other three subjects could be attributed to underlying liver injury as their GGT or ALP levels were above the reference range, although did not meet exclusion criteria (≥3x and ≥ 2x ULN, respectively). To evaluate the liver specificity of serum GLDH, we examined the correlation of serum GLDH with serum CK, a widely used biomarker of muscle injury. There was a poor correlation between GLDH and CK (Fig 1A; r_s_ = 0.39). As expected, there was a good correlation between ALT and CK (Fig 1B; r_s_ = 0.63). Taken together, these data indicate that serum GLDH levels in humans are not affected by acute muscle injury, while serum ALT levels are directly correlated to acute muscle injury.

To assess the impact of chronic muscle injury on serum levels of GLDH, we examined GLDH levels in serum samples collected from patients with DMD. Samples were collected from ambulatory DMD patients (males ages 5–14) that had previously been diagnosed based on clinical features, elevated CK levels and the absence of dystrophin expression. The GLDH levels in DMD subjects were compared with samples collected from age matched healthy male subjects (Table 1). The DMD patients exhibited extensive muscle injury with high and variable serum activity levels of ALT (~20x), AST (~9x), and creatine kinase (CK, ~74x) a commonly used biomarker of muscle damage. In contrast, serum GLDH activity was unaffected by muscle injury in DMD patients (5 ± 2 U/L) and within the normal reference range of healthy age-matched subjects (4 ± 2 U/L).

**Table 1:** GLDH levels are not affected by DMD.

Subject cohort	GLDH (U/L)	ALT (U/L)	AST (U/L)	CK (U/L)
**Healthy boys** (University of Michigan, n = 35)	4 ± 2	19 ± 7	26 ± 6	151 ± 88
**DMD patients** (University of Florida, n = 40)	5 ± 2	378 ± 214	235 ± 145	11162 ± 7977

**Fig 2:**
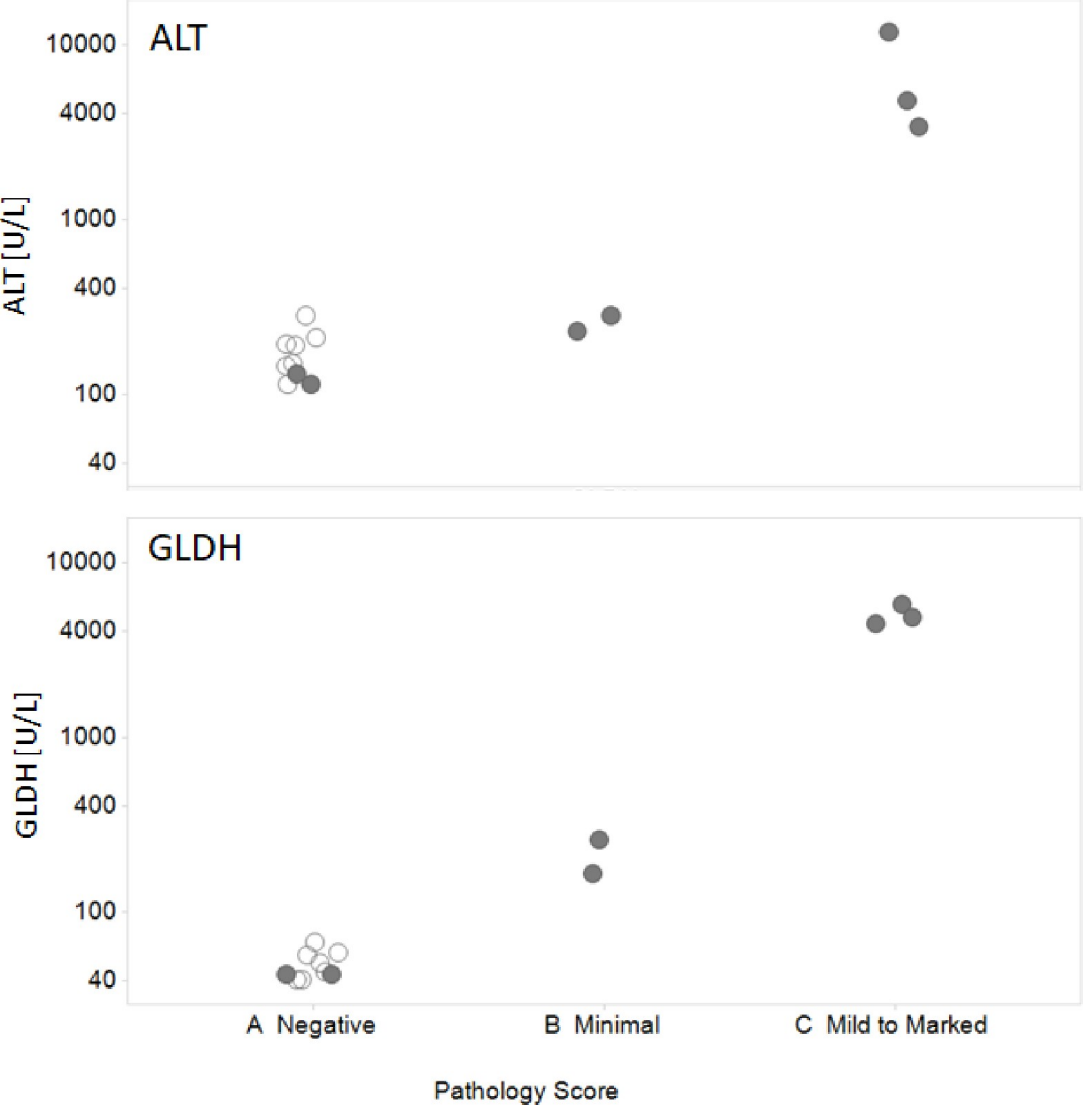
GLDH detects the onset of liver injury in a mouse model of DMD. Each data point represents ALT or GLDH serum activity of an individual mouse dosed with either vehicle (open circles) or 300 mg/kg dose APAP (shaded circles). The Pathology Score represents the degree of hepatocellular necrosis in the liver.

Furthermore, we evaluated the potential of GLDH to sensitively detect the onset of liver injury in the presence of underlying muscle disease by evaluating GLDH levels in the *mdx* mouse model of DMD. We chose this non-clinical experiment because it enabled the use of histopathology for precise characterization of liver and muscle injury given that histopathological characterization of human injury is not ethically feasible. In this study, 8 week-old *DMD*^*mdx*^, referred to as *mdx* mice, were treated with 300 mg/kg APAP a model hepatotoxicant, or vehicle control. The mice were sacrificed 24 hours after treatment. The serum was used for biomarker analysis; and liver and muscle tissues were used for histopathological analysis of liver and muscle injury (Fig 2).

All mice in this experiment displayed mild to moderate muscle pathology detected by histopathologic analysis. As expected, treatment of mice with 300 mg/kg APAP produced variable hepatotoxicity. Histopathologic analysis identified 2 non-responders (no evidence of hepatocellular necrosis), 2 mice with minimal signs of hepatocellular necrosis and 3 mice with moderate to marked centrilobular necrosis. In the case of ALT, only mice with moderate to marked liver injury had increased serum ALT activity when compared to vehicle treated mice. The ALT serum activity in two mice with only minimal liver injury was indistinguishable from 6 vehicle-treated mice and 2 non-responder APAP-treated mice. In contrast to ALT, serum GLDH activity clearly correlated with the degree of APAP-induced liver injury (Fig 2). The GLDH activity of non-responders was within normal levels for the *mdx* mice, whereas the mice with minimal liver injury showed statistically significant increases in serum GLDH activity. The GLDH increase was even more pronounced in mice with moderate and marked liver injury. In contrast to ALT, GLDH was normal within the expected reference range for 6 vehicle-treated mice and 2 non-responder APAP-treated mice.

**Fig 3:**
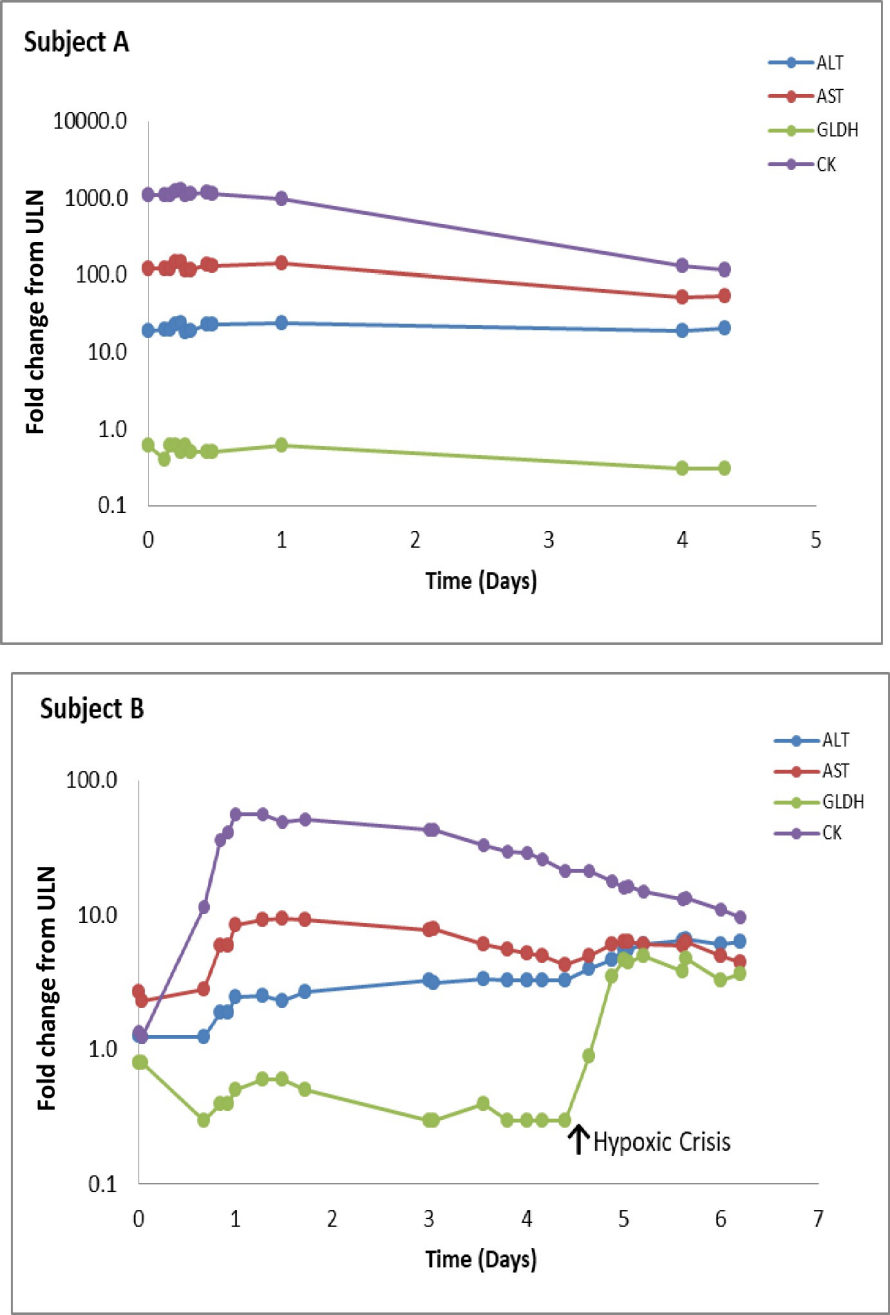
GLDH detects the onset of liver injury in a subject with rhabdomyolysis. Comparison of ALT, AST, GLDH and CK levels from consecutive blood draws for 2 subjects with rhabdomyolysis. The values represent fold increase from control using the upper limit of normal (ULN) in healthy subjects as the control level.

To demonstrate the utility of GLDH for detection of liver injury in human subjects with underlying muscle disease, we evaluated biomarker time courses in cases of rhabdomyolysis (Fig 3). At the time of diagnosis, subject A showed significant elevations of ALT, AST, and CK and showed only incremental improvement during the observation period. The fact that serum GLDH stayed within the normal enzymatic activity level during the whole observation period in this subject indicates the absence of liver injury.

The second subject (subject B) developed rhabdomyolysis characterized by a large increase in CK followed by increases in serum AST and ALT. During hospitalization, the subject suffered a severe asthmatic attack with respiratory failure requiring both ventilation and extracorporeal membrane oxygenation (ECHMO). Considering the extent of initial hypoxia, numerous concurrent medications, and the presence of hyperglycemia in conjunction with elevated amylase and lipase, we concluded that liver injury was plausible. The GLDH enzymatic activity increase in subject B correlated with onset and resolution of severe hypoxia indicating that GLDH was capable of detecting hypoxic liver damage on a background of muscle injury (Fig 3).

## Discussion

Serum enzymatic activity of ALT is used as a gold standard biomarker for diagnosis of the onset of liver injury in clinical practice. Although extremely useful, the application of ALT as a biomarker of liver injury is severely limited in subjects with underlying muscle disease due to the lack of liver specificity of the ALT enzyme. In subjects with muscle damage, the increased serum activity of ALT released from damaged muscle tissue can effectively mask the ALT activity originated from liver as a consequence of hepatocellular damage.

Several approaches have been proposed to improve the diagnosis of the onset of liver injury in subjects with underlying muscle diseases and to augment the interpretation of ALT results in clinical trials. For example, the observation that serum ALT levels show a strong correlation (r = 0.78, p < 0.0001) with the muscle specific injury biomarker creatinine kinase (CK), led to a proposal to assess liver injury in subjects with muscle disease using ALT levels correlated with CK levels [25,26]. The authors developed an equation that describes the correlation of ALT and CK and used it to predict ALT levels from measured CK levels. Liver injury is predicted when the actual/measured ALT levels are higher than the predicted levels. Although this approach is promising, the natural variability of ALT and CK levels in subjects with muscle injury, makes the data interpretation challenging.

Alternatively, serum gamma-glutamyl transferase (GGT) has been proposed as a liver specific biomarker of liver injury in subjects with underlying muscle disease. It has been reported that in DMD patients, normal serum GGT reflects the absence of liver impairments while elevated serum ALT and CK correspond to underlying muscle disease [27]. Although GGT is a sensitive biomarker of biliary epithelial damage its utility as a biomarker of hepatocellular injury is severely limited [28]. In addition, a number of confounding factors can affect GGT levels including damage to other organs, i.e. heart and pancreas, consumption of alcohol, some drug treatments, and variations due to age, race and gender [29,30]. Therefore, while this approach addresses the lack of liver specificity of ALT, the inferior performance of GGT to predict hepatocellular injury limits its impact.

GLDH is a liver specific enzyme that has shown great potential as an alternative to ALT for detection of liver injury in non-clinical species [12–18] and human studies [20–22]. Since the vast majority of GLDH is present in hepatocytes (14,15), serum GLDH activity has been proposed as a liver specific biomarker of liver injury in human subjects [19,20]. Therefore, we evaluated the potential of GLDH to diagnose liver injury in subjects with underlying muscle disease.

### Serum GLDH is not affected by muscle injury

To assess the impact of muscle impairment on serum GLDH levels in human subjects, we compared serum GLDH activity from 125 healthy subjects with the serum GLDH activity from 131 adult subjects with a wide range of acute and chronic muscle impairments and we compared the serum GLDH activity of 40 pediatric patients with DMD with age matched unaffected controls. DMD is a hereditary muscle disease that causes extensive chronic muscle injury. Since evaluating muscle damage by histology in humans for this study was not ethically feasible, we assessed the effect of muscle damage on serum ALT and GLDH by evaluating the correlation of serum CK, a widely used biomarker of muscle injury [31] with ALT and GLDH. As expected, since both ALT and CK are released from damaged muscle tissues, the serum ALT activity in subjects with muscle impairments correlated with CK (Fig 1A; r_s_ = 0.63). This result is in accord with previously published data that reported a correlation coefficient of r_s_ = 0.78 [25,26]. In contrast to ALT, serum GLDH activity did not correlate with CK in subjects with muscle impairment (Fig 1B; r_s_ = 0.39). Furthermore, serum GLDH levels in subjects with DMD with chronic muscle damage were indistinguishable from healthy age matched subjects; while ALT and AST levels were elevated 20- and 9-fold, respectively in subjects with DMD (Table 1). Although traces of GLDH have been reported in muscle tissue [4,9], our data provide clear evidence that muscle damage does not lead to a detectable increase of GLDH activity in serum in subjects with a wide variety of muscle impairments. Therefore, GLDH may have utility as a biomarker of drug induced liver injury in clinical trials of new therapies to treat muscle diseases such as DMD.

### Serum GLDH detects liver injury in the presence of underlying muscle damage

To evaluate the potential of GLDH to detect liver injury on the background of concurrent muscle damage, we used the *mdx* mouse model of DMD carrying a spontaneous nonsense mutation in exon 23 of the mouse dystrophin gene resulting in the absence of dystrophin expression [32]. These mice present with a muscular dystrophy phenotype that features skeletal muscle damage and weakness [33]. This model enabled us to reproducibly elicit concurrent muscle and liver injury that could be precisely evaluated *via* histopathological analysis. The *mdx* mice were dosed with 300 mg/kg of APAP, a model liver toxicant, for 24 hours and the extent of muscle and liver injury was detected via histopathological analysis. The degree of the resulting muscle and liver damage was compared with ALT and GLDH (Fig 2). As expected, all mice demonstrated muscle injury. The serum GLDH levels significantly increased in the presence of both minimal and/or marked liver injury and correlated well with the degree of liver injury. In contrast, ALT increases were detectable only in mice exhibiting marked liver injury (Fig 2). This experiment clearly documents the limitation of ALT for detection of liver injury in subjects with underlying muscle disease. In this situation, low grade liver injury is effectively masked by ALT originating from muscle as a consequence of muscle damage.

In order to confirm the ability of GLDH to detect liver injury on the background of muscle damage in humans, we evaluated serum samples from hospitalized patients with rhabdomyolysis (Fig 3). One patient displayed muscle damage characterized by increased ALT, AST and CK. Since this subject did not develop any signs of liver injury, GLDH remained within normal levels throughout the observation period (Subject A in Fig 3). In contrast, the second subject went through a severe asthmatic attack with respiratory failure requiring both ventilation and extracorporeal membrane oxygenation (ECHMO) before recovering. Although ALT, AST and CK levels showed a characteristic profile of rhabdomyolysis, GLDH levels exhibited marked elevation coinciding with hypoxic crisis (Subject B in Fig 3). Obtaining a biopsy for histopathologic analysis was not ethically possible. However, the medical adjudication concluded that potential liver injury was likely due to the extent of initial hypoxia, numerous concurrent medications, and hyperglycemia in conjunction with elevated amylase and lipase. GLDH was able to detect minimal centrilobular necrosis in the *mdx* mice and the increase in GLDH in the human subject coincided with hypoxia, strongly suggesting that GLDH is capable of detecting liver injury in subjects with underlying muscle damage.

## Conclusions

Lack of liver specificity confounds the use of ALT for the diagnosis of the onset of liver injury in patients with underlying muscle disease and may lead to the misdiagnosis of hepatocellular injury in patents with yet to be identified muscle disease. The current approaches for diagnosis of liver injury in patients with muscle diseases do not provide accurate assessment of hepatocellular damage and rely on the evaluation of the correlation of ALT with CK levels or GGT as an alternative to ALT. GLDH has shown promise as a sensitive and accurate biomarker of liver injury in humans [20]. In this study we show that, in contrast to ALT, serum GLDH activity is not affected by muscle damage. To our knowledge, our study is the first to demonstrate that serum GLDH can detect liver injury in the presence of concurrent muscle damage in mice and humans. GLDH has the potential to improve diagnosis of hepatocellular injury in patient populations with underlying muscle impairments and in clinical trials of new therapies for treatment of patients with muscle diseases.

## Supporting information

S1 Data (XLSX)
